# Topical ophthalmic atropine in horses, pharmacokinetics and effect on intestinal motility

**DOI:** 10.1186/s12917-021-02847-4

**Published:** 2021-04-07

**Authors:** L. Ström, F. Dalin, M. Domberg, C. Stenlund, U. Bondesson, M. Hedeland, P-L Toutain, C. Ekstrand

**Affiliations:** 1 Department of Clinical Sciences, Division of Large Animal Surgery, Swedish University of Agricultural Sciences, Uppsala, Sweden; 2 Department of Chemistry, Environment and Feed Hygiene, National Veterinary Institute, Uppsala, Sweden; 3 Department of Medicinal Chemistry, Analytical Pharmaceutical Chemistry, Uppsala University, Uppsala, Sweden; 4 INTHERES, Université de Toulouse, INRA, ENVT, Toulouse, France; 5 The Royal Veterinary College, University of London, London, UK; 6 Department of Biomedicine and Veterinary Public Health, Division of Pharmacology and Toxicology, Swedish University of Agricultural Sciences, P.O. Box 7028, SE-750 07 Uppsala, Sweden

**Keywords:** Colic, Equine, Pharmacokinetics, Pharmacodynamics, Plasma disposition, Side effect, Systemic exposure

## Abstract

**Background:**

Topical ophthalmic atropine sulfate is an important part of the treatment protocol in equine uveitis. Frequent administration of topical atropine may cause decreased intestinal motility and colic in horses due to systemic exposure. Atropine pharmacokinetics are unknown in horses and this knowledge gap could impede the use of atropine because of the presumed risk of unwanted effects. Additional information could therefore increase safety in atropine treatment.

**Results:**

Atropine sulfate (1 mg) was administered in two experiments: In part I, atropine sulfate was administered intravenously and topically (manually as eye drops and through a subpalpebral lavage system) to six horses to document atropine disposition. Blood-samples were collected regularly and plasma was analyzed for atropine using UHPLC-MS/MS. Atropine plasma concentration was below lower limit of quantification (0.05 μg/L) within five hours, after both topical and IV administration. Atropine data were analyzed by means of population compartmental modeling and pharmacokinetic parameters estimated. The typical value was 1.7 L/kg for the steady-state volume of distribution. Total plasma clearance was 1.9 L/h‧kg. The bioavailability after administration of an ophthalmic preparation as an eye drop or topical infusion were 69 and 68%, respectively. The terminal half-life was short (0.8 h). In part II, topical ophthalmic atropine sulfate and control treatment was administered to four horses in two dosing regimens to assess the effect on gastro-intestinal motility. Borborygmi-frequency monitored by auscultation was used for estimation of gut motility. A statistically significant decrease in intestinal motility was observed after administration of 1 mg topical ophthalmic atropine sulfate every three hours compared to control, but not after administration every six hours. Clinical signs of colic were not observed under any of the treatment protocols.

**Conclusions:**

Taking the plasma exposure after topical administration into consideration, data and simulations indicate that eye drops administrated at a one and three hour interval will lead to atropine accumulation in plasma over 24 h but that a six hour interval allows total washout of atropine between two topical administrations. If constant corneal and conjunctival atropine exposure is required, a topical constant rate infusion at 5 μg/kg/24 h offers a safe alternative.

## Background

Equine uveitis is a leading cause of blindness in horses [1–3]. Uveitis may cause ciliary muscle spasm as well as pupillary contraction (miosis). The spasm is painful and chronic complications may occur, including synechia between tissues in the eye that can cause persistent pupil constriction, glaucoma and decreased vision. Topical ophthalmic administration of the non-selective muscarinic receptor-antagonist atropine sulfate induce cycloplegia, which alleviates the painful spasm caused by uveitis. Atropine also induces mydriasis, which is beneficial to minimize the risk for the development of synechia. Synechia can obstruct aqueous humor outflow and thus cause secondary glaucoma, as well as cause reduced vision and blindness through persistent pupil constriction [4, 5]. Atropine has also been shown to stabilize the blood-aqueous barrier, and thereby reduce the leakage of detrimental inflammatory cells and debris into the aqueous chamber [6]. Thus, atropine is an important part of the treatment protocol in equine uveitis [7, 8]. Unfortunately, intestinal motility is also mediated by muscarinic receptors, and systemic atropine exposure has been shown to decrease borborygmi-frequency, increase intestinal transit time, and has also been associated with colic in horses [6, 9–15]. After topical administration of 1 mg atropine sulfate every hour in the conjunctival sac, intestinal motility decreased and clinical signs of abdominal pain developed in 4/6 horses [16]. The onset of these adverse effects was between 11 h and 22 h after the first administered dose. In contrast, 1 mg of topical ophthalmic atropine sulfate every six hours did not affect intestinal transit time or borborygmi-frequency, nor cause signs of colic or abdominal discomfort in another study in six horses [17]. These conflicting results suggest that the systemic atropine exposure after the less frequent dosing-protocol was not associated with a decrease in intestinal motility. This hypothesis was also supported by non-detectable plasma concentrations of atropine six hours after topical administration of 1 mg atropine sulfate, which was the first sampling time post administration [17]. However, the systemic disposition of atropine in horses following different modalities of atropine administration including topical eye medication is currently not reported in horses. The aims of this study was to characterize, to model, and to simulate the pharmacokinetics of atropine in horses. A second aim was to investigate the borborygmi-frequency response to 1 mg atropine sulfate administered topically following different dosing regimens. The third aim was to relate the simulated atropine plasma concentration-time courses to the gastrointestinal motility response and development of signs of colic.

## Results

### Part I

#### Plasma exposure of atropine

**Fig. 1:**
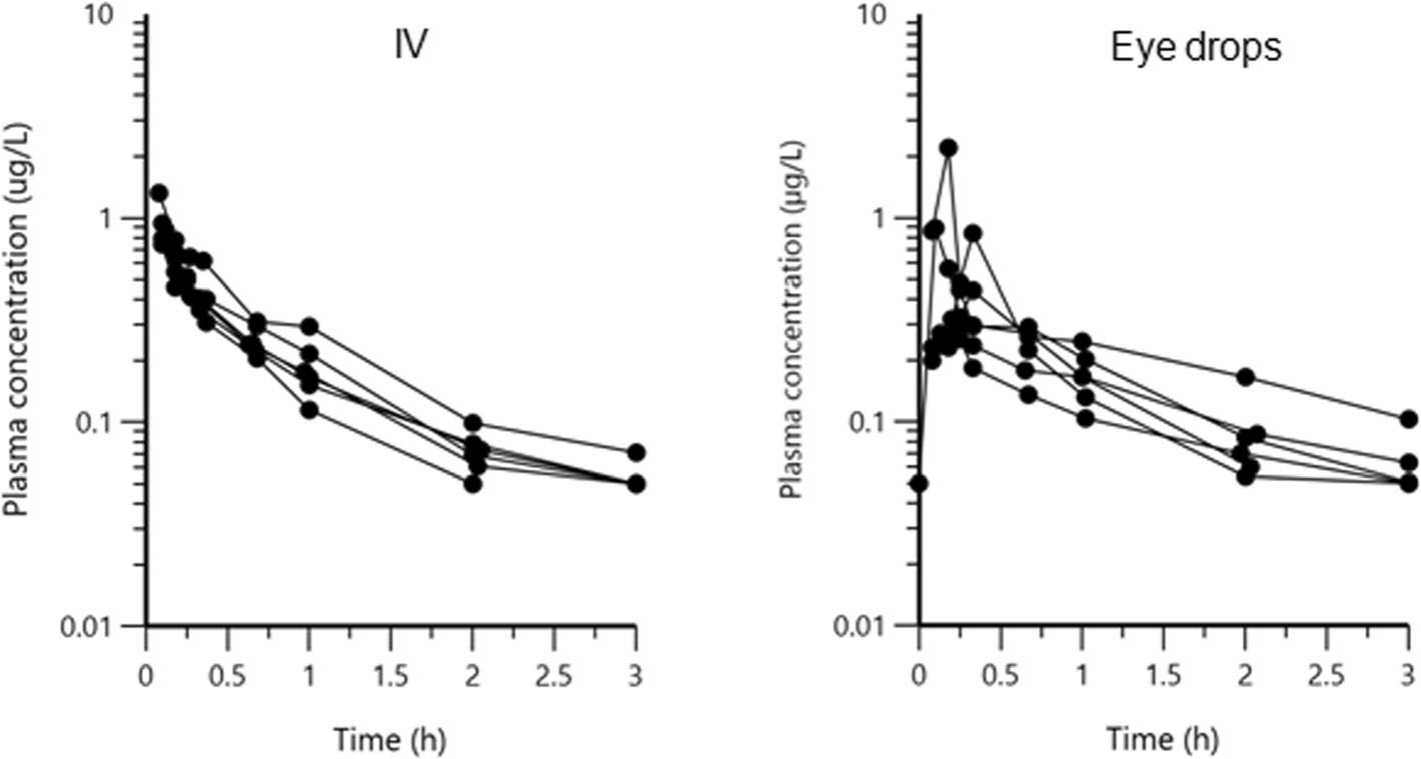
Semi-logarithmic spaghetti plots of the plasma disposition curves of atropine over 3 h after a single dose administration of atropine as 1 mg atropine sulfate as an intravenous (IV) bolus dose (left plot) and as a bolus dose (same dose as for IV with a 0.1 mL volume) administered topically using an ophthalmic solution (eye drops) (right plot). The lower limit of quantification for the analytical method was 0.05 μg/L.

**Fig. 2:**
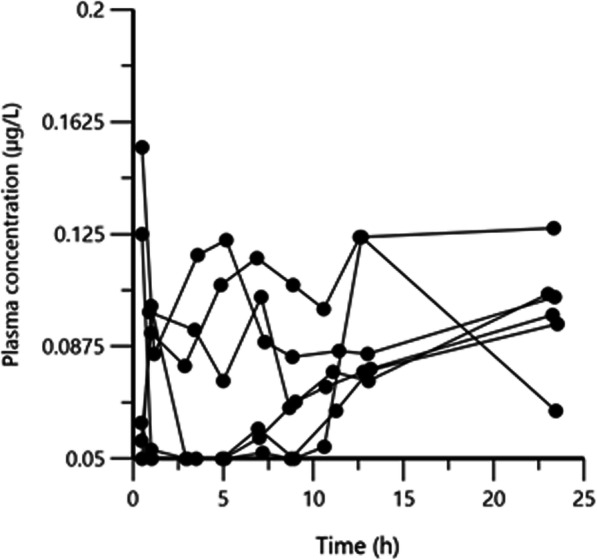
Arithmetic spaghetti plot of the disposition curves of atropine over 24 h after administration of 0.14 mg/h atropine sulfate topically as constant rate infusion using a subpalpebral lavage system over 24 h in six horses. The lower limit of quantification for the analytical method was 0.05 μg/L.

After intravenous (IV) administration of 1 mg atropine sulfate (corresponding to 0.835 mg atropine) as a bolus dose, the plasma atropine concentrations decreased in a rather regular pattern and fell below lower limit of quantification (LOQ, 0.05 μg/L) between one to five hours after drug administration (Fig. 1). After a single dose of 1 mg atropine sulfate administered as a topical ophthalmic solution (eye drops) in the conjunctival sac, the plasma atropine concentration was of the same order of magnitude as after the IV administration (Fig. 1). The absorption was rapid with a peak plasma concentration being observed within the first 30 min and plasma concentrations fell below LOQ two or three hours after atropine administration. During administration of 0.14 mg/h atropine sulfate as subpalpebral constant rate infusion using a subpalpebral lavage system (SPL), atropine plasma concentration-time curves were largely scattered around 0.1 μg/L and suggesting more variability than after the eye drop administration (Fig. 2).

#### Pharmacokinetics of atropine

**Fig. 3:**
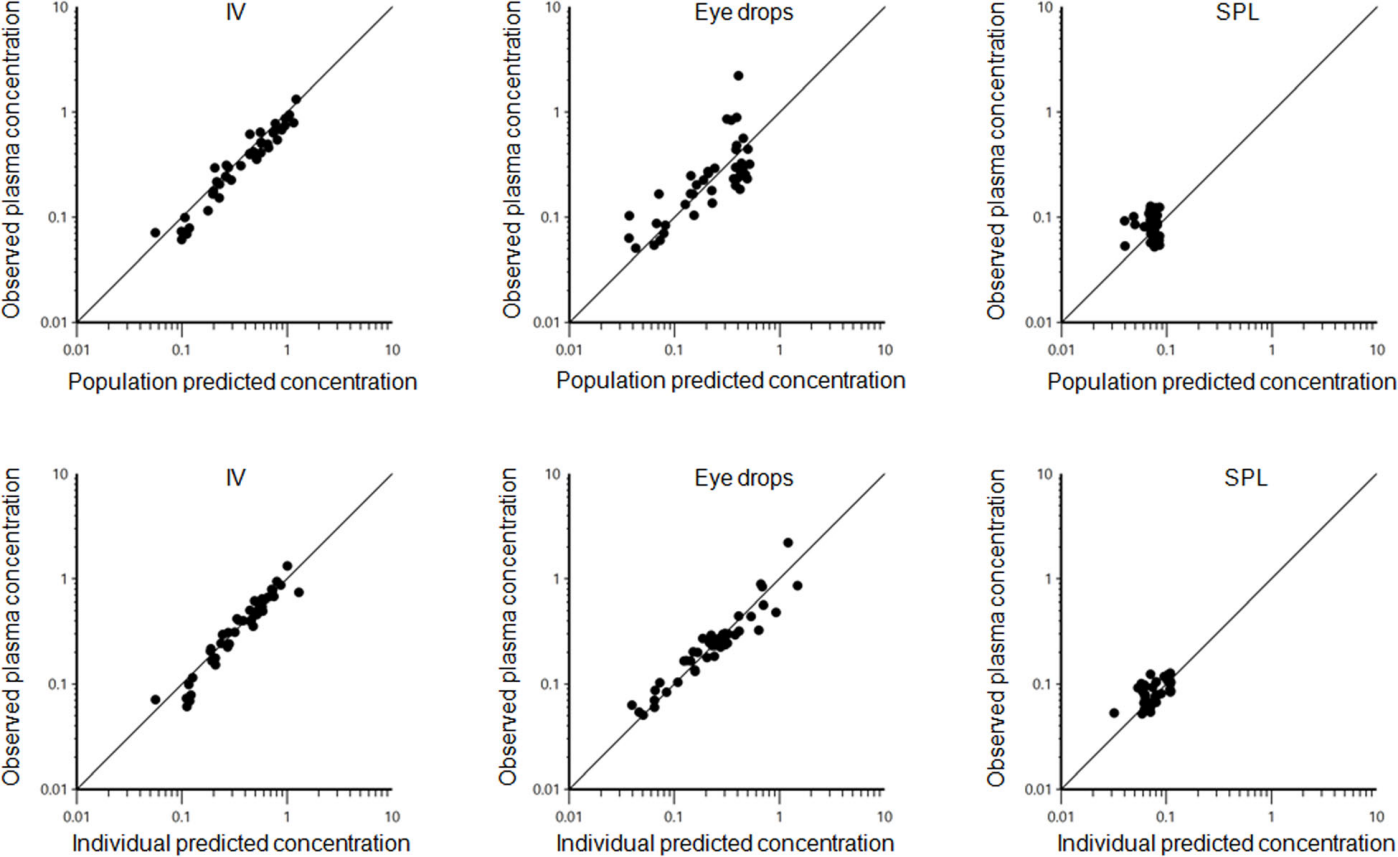
Logarithmic plots of observed atropine plasma concentrations vs. populations predicted (upper plots) and individual predicted atropine plasma concentrations (lower plots) for the single intravenous administration (IV) (left plots), single administration of an ophthalmic solution (eye drops) (middle plots) and 24 h constant rate infusion using a subpalpebral lavage system (SPL) (right plot). All concentrations are given in μg/L. Data were evenly distributed about the line of unity for the IV, eye drops administration and the 24 h SPL-infusion indicating no major bias in neither the population component nor the random component of the model

**Fig. 4:**
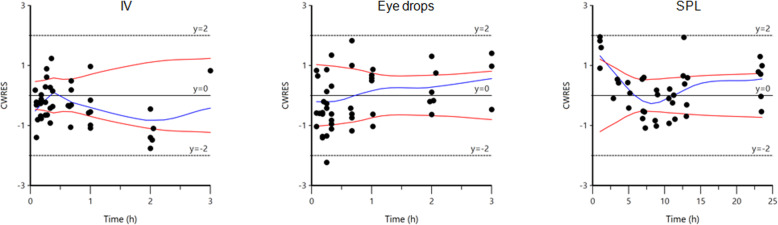
Scatter plot of the CWRES (conditional weighted residuals) vs. time (h) for the single intravenous administration (IV) administration (left panel), single administration of an ophthalmic solution (eye drops) (middle panel) and constant rate infusion using a subpalpebral lavage system (SPL) (right panel)

**Fig. 5:**
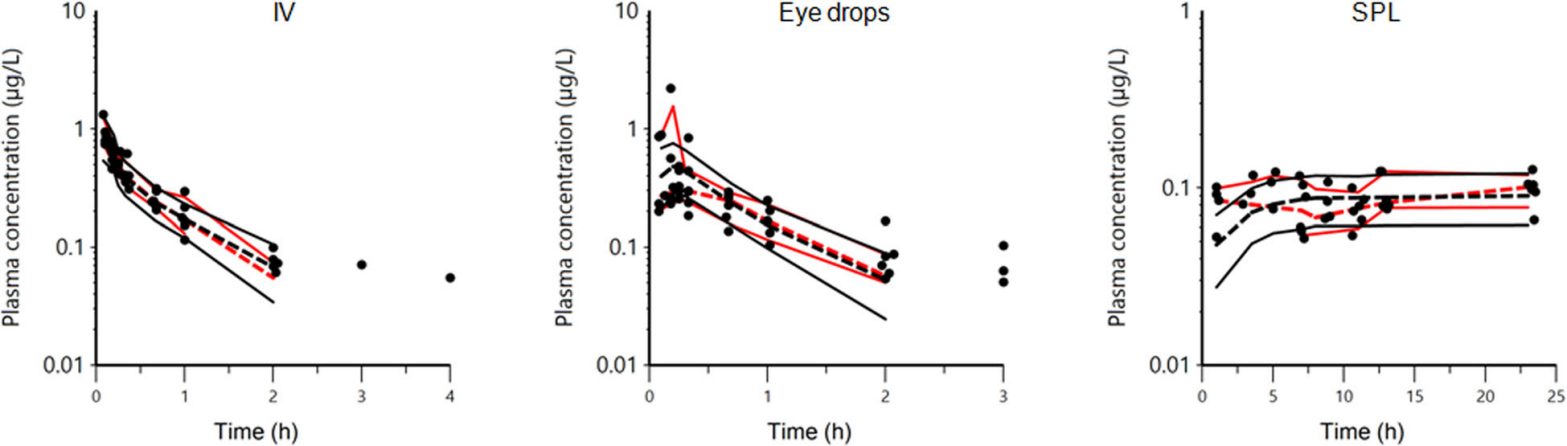
Visual Prediction Check according to route of administration: single intravenous administration (IV) (left), single administration of an ophthalmic solution (eye drops) (middle) and constant rate infusion using a subpalpebral lavage system (SPL) (right). The empirical and predicted 20th and 80th percentiles are shown in solid red and black lines respectively. The observed and predicted 50th percentile (median) are shown in red and black broken lines respectively. The black dots are observed data. A binning option with explicit centers was used to generate these plots

Model evaluation (goodness of fit plots and Akaike Information Criterion) indicated the appropriateness of a two compartment structural model, of its random component and of the selected residual error model. Scatter plots for observed concentrations versus predicted concentration (PRED) obtained by solving the structural model with typical values of its parameters and scatter plot of observed concentrations vs. individual predicted concentration (IPRED) obtained by solving the model with individual parameters i.e. with their post-hoc values are given in Fig. 3. Plot of conditional weighted residual (CWRES) over time are given in Fig. 4. Inspection of Fig. 4 show that residues were randomly scattered around zero which is support for the selection of the residual error model. Visual Predictive Check (VPC) to assess whether the current model describes the central tendency and variability in the observed data by comparing the observed median and quantiles to the median and quantiles of the simulated dataset is given in Fig. 5. Inspection of VPC indicates that the full model was able to replicate the central tendency and variation in observed data.

**Fig. 6:**
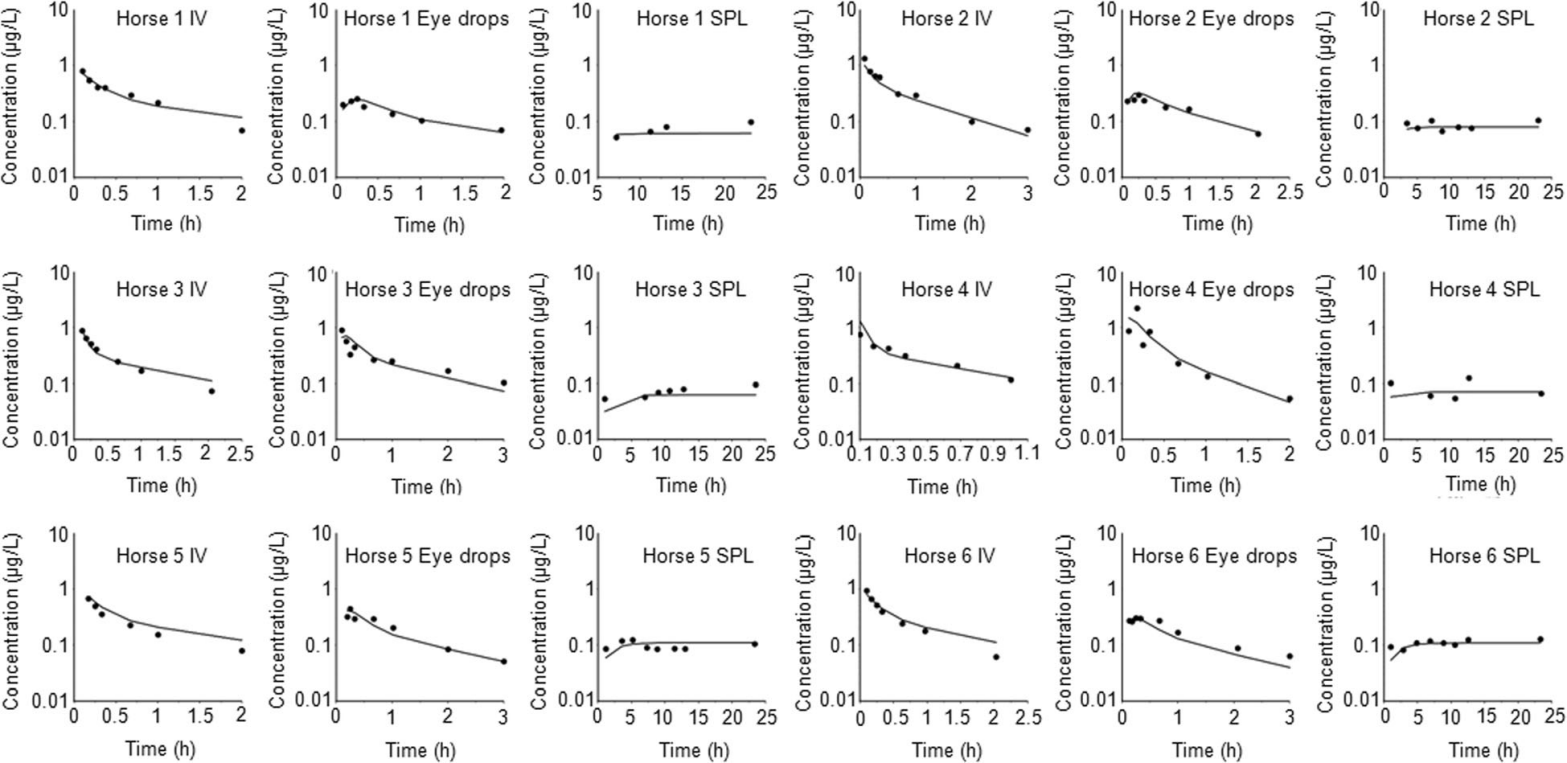
Observed (symbols) and individual predicted (lines) plasma concentration-time courses for the six horses and the three modalities (single intravenous administration (IV), single administration of an ophthalmic solution (eye-drops) and ophthalmic solusion administered as constant rate infusion using a subpalpebral lavage system (SPL)) of atropine administration

The individual predicted and observed plasma concentration for the six horses and the three modalities of administration are given in Fig. 6. Visual inspection of the 18 plots indicates that the selected model was able to predict individual disposition curves for the three modalities of administration.

Bootstrap estimates of typical values of the primary structural parameters of the model (thetas), the secondary parameters (steady state volume of distribution, mean residence time, half-life and bioavailability) and their associated coefficient of variation as a measure of precision of their estimation are given in Table 1. The inter-animal variability values and individual post-hoc values are given in Table 2.

**Table 1:** Bootstrap estimates of structural parameters of the 2-compartment model and secondary derived parameters

	Units	Median	CV%	2.50%	97.50%
**Primary structural Parameters**					
tvV_c_	L/kg	0.646	28.03	0.314	0.803
tvV_t_	L/kg	1.148	16.39	0.740	1.277
tvCl	L/kg/h	1.905	6.63	1.771	2.148
tvCl_d_	L/kg/h	2.477	23.87	1.689	3.652
tvfdrop (ilogit, eye drop)	Scalar	0.781	172.66	0.196	324.616
tvfINF (ilogit, infusion)	Scalar	0.738	199.51	0.157	12.979
tvCMultStdev (residual, proportional)	Scalar	0.243	43.96	0.037	0.370
tvK_a_	1/h	5.948	198.88	3.238	122.226
stdev0 (residual, additive)	μg/L	0.019	42.28	0.004	0.029
**Secondary parameters**					
Bioavailability Drop	Scalar (0–1)	0.685	26.12	0.549	1.000
Bioavailability infusion	Scalar (0–1)	0.676	19.59	0.539	0.952
Half-life_absorption	h	0.117	53.82	0.019	0.226
Half-life_Beta (terminal phase)	h	0.798	12.77	0.603	0.889
Half-life_alpha (initial phase)	h	0.077	20.77	0.049	0.099
V_ss_ (steady-state volume of distribution)	L/kg	1.747	15.99	1.169	1.960
MRT (Mean residence time (IV)	h	0.884	16.16	0.617	1.050

*V*_*c*_ *and V*_*t*_ *are the volume of the central and peripheral compartment, Cl is the clearance and Cl*_*d*_ *is the intercompartmental distribution clearance, k*_*a*_ *is the absorption rate constant, fdrop and finf are the ilogit of bioavailability associated to eye drops and infusion administration, respectively. CMultStdev corresponds to the proportional component of the residual error with a value of 24.3% and stdev is the additive component of the residual. All these parameters were estimated with a reasonable precision but for the two ilogit estimates. This was due to the ilogit transformation as indicated by a rather good precision of the corresponding bioavailability (68.5 and 67.6% for the eyes drop and the infusion respectively, an ilogit value higher than 3 corresponds to a near total bioavailability.*

**Table 2:** Inter-animal variability for the different estimated structural parameters of the disposition model of atropine and individual post-hoc values

Parameters	*V*_*c*_	*Cl*	*V*_*t*_	*Cl*_*d*_	F Drop (ilogit)	F INF (ilogit)
ETA variance	0.633	0.013	0.213	0.261	7.606	1.123
Shrinkage	0.025	0.253	0.184	0.415	0.364	0.199
IIV (%)	93.95	11.23	48.70	54.60	4483.43	143.98
Individual Post-hoc values						
Horse	*V*_*c*_	*Cl*	*V*_*t*_	*Cl*_*d*_	F Drop	F INF
1	1.862	2.167	1.864	2.312	0.526	0.631
2	1.202	1.780	1.177	2.311	0.517	0.702
3	0.692	1.878	1.892	3.886	0.995	0.651
4	0.200	1.910	0.633	1.422	0.999	0.599
5	1.705	2.248	1.542	2.250	0.731	0.946
6	1.524	1.887	1.580	1.917	0.644	0.977

*ETA variance is the estimated variance of the random component of the model and IIV% is the corresponding inter-individual variability calculated from the random components of the model by means of eq.* *2**. Shrinkage was calculated with eq.* *3**and value IIV% obtained when shrinkage is > 0.3 should be regarded with caution. Post-hoc individual values were obtained by solving the population model with its fixed and random components. Vc, Vt, Cl, Cld are defined in* Table 1*. F drop and F infusion are the bioavailability associated to eye drops and infusion administration respectively*

#### Simulation of atropine exposure after different dosing regimens of topical ophthalmic solution

**Fig. 7:**
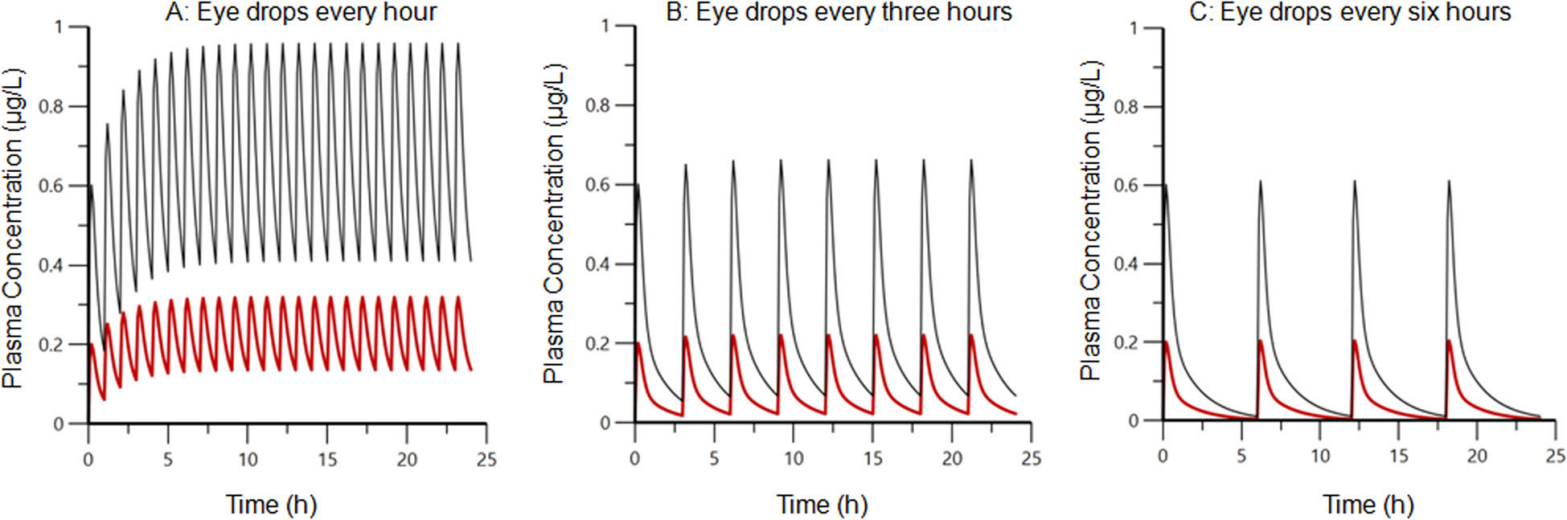
Simulation of plasma atropine concentrations following administration of either 0.5 μg/kg (red lines, mimicking the dose delivered in one drop by a dropper bottle) or 1.5 μg/kg (black lines, mimicking the dose atropine sulfate administered in this study (1 mg)) every hour (left plot, **a**), every three hours (middle plot, **b**) or every six hours (right plot, **c**)

Plasma atropine concentration-time courses after different dosing regimens of topical ophthalmic administration were simulated and are shown in Fig. 7. Visual inspection of Fig. 7 indicates that administration of eye drops at 1 h intervals for 24 h will lead to atropine accumulation with plasma concentrations oscillating between 0.1 and 0.3 μg/L after the dose 0.5 μg/kg (total dose of 12 μg/kg over 24 h). The dose 0.5 μg/kg mimicked the dose delivered in one drop by a dropper bottle. Eye drops at one hour interval for 24 h will lead to atropine accumulation with plasma concentrations oscillating between 0.4 and 0.9 μg/L after the dose 1.5 μg/kg (total dose 36 μg/kg over 24 h). The dose 1.5 μg/kg mimicked the dose administered in this study and by Williams et al., [16]. When the 0.5 μg/kg and 1.5 μg/kg doses were simulated for dosing every three hours, findings indicated that atropine would continue to accumulate, but to a lesser extent and atropine exposure would be lower. With dosing every six hours accumulation of atropine in plasma would no longer occur.

**Fig. 8:**
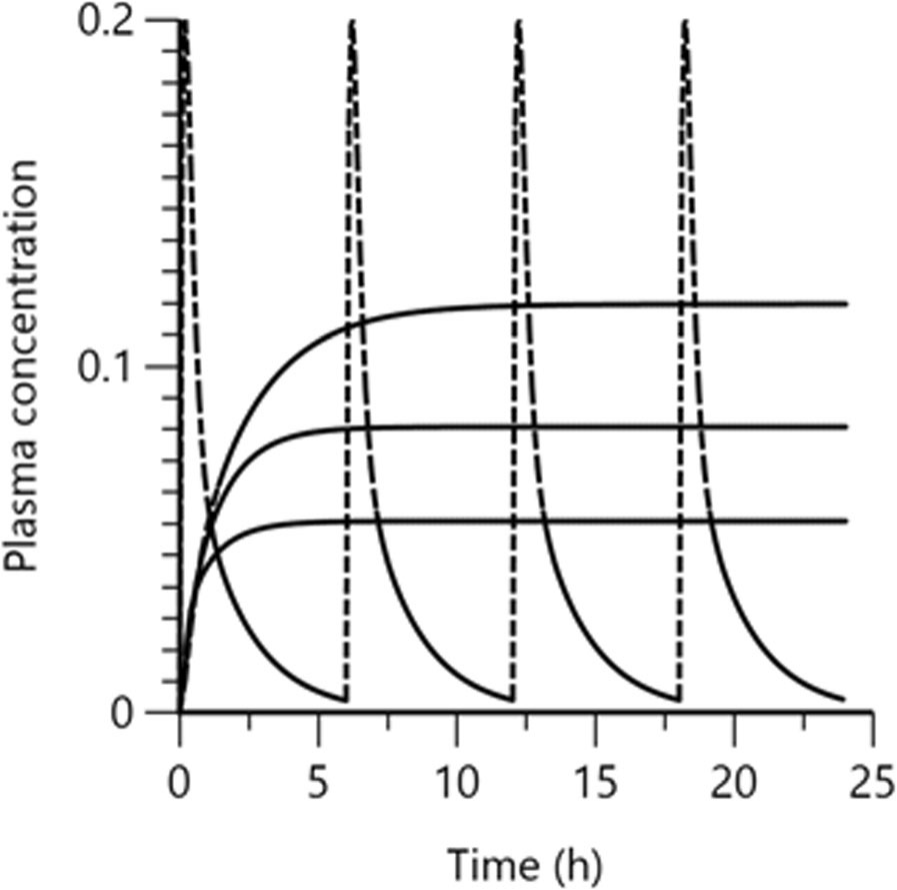
Prediction interval (90%) for plasma exposure following a constant rate infusion using a subpalpebral lavage system (SPL) at 5 μg/kg per 24 h vs. a series of administered topical ophthalmic solution (eye drops) at 0.5 μg/kg at 6 h intervals. Solid horizontal lines represent the 5, 50 and 95th quantile of a population of 1000 horses obtained by Monte Carlo Simulation with 90% of horses ranging between average plasma concentrations of 0.053 μ/L (lower solid line) and 0.108 μ/L (upper solid line). Broken line corresponds to simulated atropine plasma concentration-time course after 0.5 μg/kg atropine administered as topical ophthalmic solution at 6 h interval. The simulation was based on typical values of the primary structural parameters of the model (thetas)

Using Monte Carlo simulation, we simulated a population of 1000 horses for a subpalpebral constant rate infusion through an SPL of 5 μg/kg over 24 h (which correspond to 24 h atropine sulfate infusion at the rate 0.14 mg/h) to assess influence of inter-animal variability on the achieved steady-state plasma concentration. We computed that the steady-state plasma concentration for 90% of horses (i.e. the prediction interval) was between 0.053 and 0.108 μg/L (Fig. 8).

### Part II

**Fig. 9:**
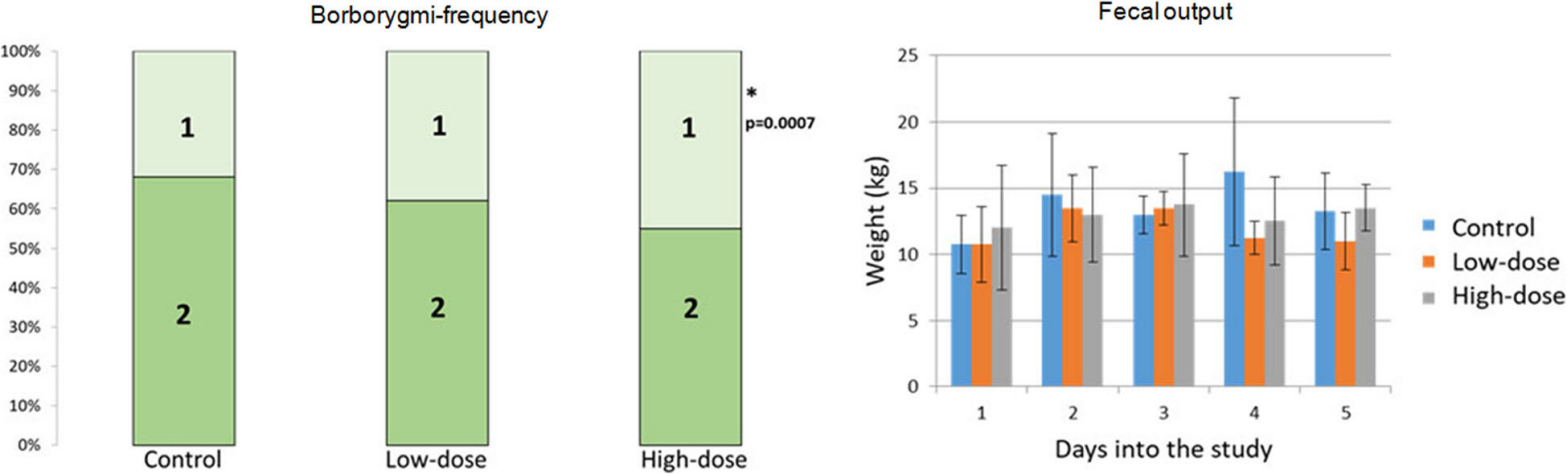
Borborygmi-frequencies (left) and fecal output (right) during administration of eye drops at high-dose (0.1 mL 1% atropine sulfate solution every three hours), low-dose (0.1 mL 1% atropine sulfate solution every six hours) and control regimens to four horses. A significant decrease in borborygmi-frequency (scored as follows: 1 = intermittent gut sounds and 2 = continuous gut sounds over the auscultation period) was observed during the high-dose regimen compared to control (*p* = 0.0007), but not when during the low-dose regimen (*p* = 0.17). There were no significant differences in fecal output between studied protocols (*p* = 0.72). The graph display the average fecal weight (kg) per day and maximum and minimum range during each protocol

Eye drops (0.1 mL of 1% atropine sulfate solution corresponding to 0.835 mg atropine) was administered every three hours (high dose), every six hours (low dose) and saline (control), in a cross over study including four horses. None of the horses showed any signs of abdominal pain during the study periods. Pupils were fully dilated in the treated eye of all horses when atropine was administered, whereas they were fully responsive to light during the control treatment. Gut sounds (i.e. score 1 = intermittent sounds or 2 = continuous sounds) were present at every auscultation point in all horses (in total 1008 observations). Figure 9 shows the overall results from evaluation of borborygmi-frequency and fecal output. A statistically significant decrease in gut sound scores was observed during the high dose regime (*p* = 0.0007) compared to control treatment, but not during the low dose regime (*p* = 0.17). No significant difference was seen between control and treatment regimens when comparing heart rate (*p =* 0.17), respiratory rate (*p* = 0.83) or fecal output (*p* = 0.72).

## Discussion

In order to better understand the relationship between atropine absorption and systemic exposure following eye medication (topical ophthalmic eye drops and through an SPL) and effects on the gastrointestinal system, we conducted an PK study to quantify basic pharmacokinetic parameters of atropine in horses. The short half-life of atropine in horses (less than one hour) and the rapid washout of plasma atropine between two administered eye drops as illustrated by the simulation (Fig. 7), can be explained by the high atropine clearance (Table 1). After topical atropine eye drop administration in horses, the maximum plasma atropine concentration was observed within 15 min in all horses with a typical half-life of absorption of 11 min. Ophthalmic solutions are generally available in dropper bottles that deliver drops with a volume that ranges from 25 μL to 70 μL (average 40 μL) [18]. With larger volumes, a risk of adverse systemic effects due to drainage of the excess volume through the nasolacrimal canal and increased systemic uptake over the nasal/buccal mucus membranes can be hypothesized, assuming that the entire dose given is deposited in the eye. In horses the tear volume is 360 μL [19]. Consequently it was assumed that the lower volume that was administered (100 μL) using eye drops in the present trial was deposited to the eye. Regardless the site of absorption (conjunctiva, nasolacrimal canal etc.), the extent of bioavailability was close to 70% indicating that most of the administered atropine dose was absorbed and thus made systemically available for possible adverse drug reactions.

The statistically significant decreased borborygmi-frequency observed after topical administration of 1 mg atropine sulfate every third hour compared to control (*p* = 0.0007) in part II of this study, indicate that the systemic exposure of atropine was sufficient to inhibit gut motility at the high-dose regimen. However, none of the horses developed signs of colic and gut sounds were present at all auscultation points. After topical administration of 0.1 mL of 1% atropine ophthalmic solution (corresponding to the dose 1 mg atropine sulfate) every six hours, there was no significant difference in borborygmi-frequency compared to placebo (*p* = 0.17), which is consistent with an earlier report [17]. Moreover, neither the present study nor the study by Wehrman et al. [17] detected any signs of abdominal pain. In contrast, topical administration of 1 mg atropine sulfate every hour (i.e. 2 μg/kg BW per hour) has been shown to induce colic in horses [16]. These differences are most likely due to the different dosing protocols. Due to the short half-life for atropine in plasma, adopting the six-hour dose administration interval allowed a partial to total elimination of atropine exposure between two administrations, while there was substantial atropine accumulation with the one hour interval dosing protocol as illustrated by simulations presented in Fig. 7. According to our simulations, the hourly eye drop dosing regimen associated with gastrointestinal discomfort [16] will result in atropine accumulation in plasma, with peak concentrations above 0.9 μg/L and trough concentrations around 0.4 μg/L. However, decreasing the hourly dose to 0.5 μg/kg (mimicking the use of a droplet bottle) decrease peak and trough concentration to approximately 0.3 and 0.15 μg/mL, respectively. For the dosage regimens 0.5 μg/kg every three or six hours, and after topical infusion, peak plasma concentration never exceed 0.2 μg/L. For the dosage interval of three hours, some atropine accumulation, that can be evidenced by trough concentrations of about 0.03 μg/L, will likely occur at the dose 1.5 μg/kg (mimicking 0.1 mL 1% atropine sulfate solution). The simulated increased plasma exposure due to accumulation may explain why atropine eye drops every 3 h was also associated with an effect on the gastrointestinal tract (in terms of gut motility) in part II of the present study. According to our data and simulations, when using eye drops, accumulation of atropine in plasma will be totally absent at a dosage interval of six hours, which in turn avoids adverse effects on the gastro-intestinal tract as shown in part II of the present study and the study by Wehrman et al. [17].

To the best of our knowledge, there is no quantitative PD information describing atropine concentrations inhibiting gastrointestinal motility. However, the PK/PD relationship for atropine was investigated in man using heart rate and saliva flow as endpoints [20, 21]. The efficacious plasma concentration allowing to achieve half maximum effect (*EC*_*50*_) was estimated to be 3.7 and 6.2 μg/L for saliva flow and heart rate respectively. An even higher value of 15 μg/L was reported for stimulatory effects on heart rate in rats [22]. This is about 10 to 20-fold higher than the plasma concentrations observed in the present study, suggesting that the potency of atropine for inhibiting gastrointestinal motility effect in horses is much higher (meaning that the concentration to achieve half maximum effect is lower) than the reported potency for cardiovascular or secretory effects in man or rodents. This was surprising since in general larger doses are required to inhibit gastro-intestinal and urinary tract smooth muscles than inhibition of salivary secretion or vagal tone in other species [23]. In addition, the dose of atropine able to inhibit the electromyographic colonic activity in horses was reported to 100 μg/kg [24], i.e. a dose considerably higher than doses used for ophthalmic conditions. Williams et al., [16] reported that with topical administration of 1 mg atropine sulfate hourly clinical signs of abdominal pain in horses developed after 11 to 22 h. This also suggests that some accumulation (atropine or/and its metabolites) could be involved in the triggering of gastrointestinal adverse effects in horses using frequent atropine ophthalmic administration protocols. Alternatively, apart from a direct and immediate atropine effect on intestinal motility severe gastrointestinal effects could result from several other cumulating effects over time, such as mild inhibition of the gastrointestinal motility, reduction of salivary and other gastro-intestinal secretions, pain, or decreased exercise.

In horses, the atropine plasma clearance expressed per kg BW presented here (1.9 L/kg/·h) must be considered as a high clearance [25]. Disposition of high clearance drugs are often influenced by blood-flow and alteration of plasma clearance (and hence of systemic plasma atropine exposure) can be anticipated when the cardiac output or tissue perfusion are altered. A clinical relevant consequence could be increased plasma exposure due to decreased clearance caused by co-administration of drugs, for example sedation with alpha-2 receptor agonists that reduce cardiac output [26, 27]. Together with other risk factors for colic (e.g. environmental stress, pain, decreased exercise) frequent sedation of atropine treated horses in clinical settings might put the horse at risk. Additional experimental data are however required to evaluate influence of blood flow on atropine clearance.

When the drug was administered through an SPL at a dose of about 5 to 5.5 μg/Kg over 24 h by means of a constant rate infusion, atropine plasma concentrations were lower but more sustained than after topical administration (Fig. 2), due to the low input rate associated to the infusion. Together with previously presented data, our results suggest lower risk for colic after infusion compared with topical treatment every hour [16, 17]. More precisely, it is likely that if atropine plasma concentration does not exceed 0.1 μg/L (Fig. 8) adverse gastrointestinal effects are unlikely, and this infusion modality of atropine administration should be considered when frequent administration is indicated.

For the infusion, the initial data interpretation raised the main issue of variability of plasma concentrations (Fig. 2). In order to explore consequences of that variation, the model was used to simulate a population of 1000 horses for a dose of 5 μg/Kg over 24 h (Fig. 8). An acceptable prediction interval was observed, and most of the horses being located between steady-state plasma concentration of 0.05 and 0.11 μg/L which was considered acceptable in relation to the exposure assumed decreasing borborygmi-frequency based on data and simulations in this study and the studies by Williams et al. [16] and Wherman et al. [17].

## Conclusions

This study demonstrated that atropine was rapidly cleared from plasma and consequently the half-life in plasma was short. After topical administration to the tear film, there was a rapid uptake to plasma. After administration of 1 mg atropine sulfate, the atropine plasma concentration was lower than LOQ (0.05 μg/L) within four hours, after both topical and IV administration. Topical atropine had the potential to decrease intestinal motility (measured as borborygmi-frequency) with a three-hour dosing regimen. However, signs of colic were not observed in any of the evaluated treatment protocols. Nonetheless, clinical dosage regimen recommendations with no or negligible risk for colic remain to be firmly determined. However, taking the plasma exposure after topical administration into consideration, data and simulations indicated that eye drops administrated at a one hour interval will lead to a sustained atropine accumulation in plasma over 24 h, but that a six hour interval allow a total washout of atropine between two topical administrations. Finally, if multiple administrations are required to achieve constant corneal and conjunctival atropine exposure, an infusion at 5 μg/kg/24 h offers a safe alternative.

## Methods

### Horses

In part I, six Standardbred horses (four mares and two geldings) aged 7–16 year and weighing 502–642 kg were used. In part II, four Standardbred horses (two mares and two geldings) 5–13 years old and weighing 514–653 kg were used. All horses were without remarks on ophthalmic examination. Horses were kept in individual boxes and fed hay during the experimental study periods. Water was available ad libitum. The horses were exercised by hand for ten minutes three times per day (8 a.m. 2 p.m. and 8 p.m.). Between experimental periods, horses were on pasture or in paddocks during the day and in individual boxes during nights.

### Preparation

Before treatment, the hair over the jugular veins was clipped and a lidocaine + prilocaine cream (EMLA® 25 mg/g + 25 mg/g, Astra Zeneca AB, Södertälje, Sweden) was applied on the skin. One intravenous catheter (14 Ga [13 cm] Milacath, Mila International Inc., Erlanger, USA) was placed in each jugular vein and secured with three sutures. One catheter was used for atropine administration and one for collection of blood samples. The day before administration by topical infusion, horses were sedated using 0.01 mg/kg detomidine (Domosedan vet 10 mg/mL, Orion pharma animal health, Danderyd, Sweden), nerve blocks (*N. palpebralis* and *n*. supraorbitalis) were performed using 3 ml of mepivacain (Carbocain 10 mg/mL, Aspen Nordic, Ballerup, Denmark). Topical corneal anesthesia (Tetracain 1%, Bausch & Lomb Stockholm, Sweden) was induced. A subpalpebral lavage system (SPL) (Subpalpebral eye lavage kit, 36in, Mila International Inc., Erlanger, USA) was placed under the upper eyelid and was secured with four sutures. An infusion pump (Infu-Disk, administration pump, three-day delivery system, 0.14 ml/h, Mila International Inc., Erlanger, USA), prepared with 1 ml of atropine sulfate (Isopto-atropin, ophthalmic drops, 1%, Alcon Nordic), 4 ml of chloramphenicol (Kloramfenikol, ophthalmic drops, 5 mg/ml, CCS Healthcare AB, Borlänge) and 5 ml of saline (Natriumklorid,, 9 mg/ml, Fresenius Kabi, Sweden), was attached to the SPL the next morning at the start of the study.

### Monitoring

The horses were monitored every three hours during each part of the study (part I and II). One investigator performed a physical examination which included evaluation of mucous membranes, capillary refill time, pupillary light reflex (direct and indirect), menace response, heart rate and respiratory rate. A masked investigator monitored the horses for signs of abdominal pain (e.g. lack of appetite, flank watching, kicking, pawing and rolling) and performed auscultation of borborygmi (the intestinal sounds were used as a surrogate for intestinal motility which in turn predisposes to development of colic) throughout each study. For auscultation of borborygmi, four abdominal quadrants were auscultated (right and left ventral and dorsal flank) during one minute each. Each quadrant was graded from 0 to 2 (0 = no gut sounds, 1 = gut sounds during parts of the auscultation period, 2 = gut sounds during the entire auscultation period). During part II of the study, fecal output (measured as weight) was also measured daily.

### Experimental design

Part I: The horses were given atropine sulfate in a three-treatment crossover design; single IV administration, a single administration of ophthalmic solution (eye drops) and topical constant rate infusion in the eye using a subpalpebral lavage system (SPL), with a three-week washout period between treatments. For the IV-treatment, 1 mg atropine sulfate (equivalent to 0.835 mg atropine) in an injectable solution was administered (Atropin Mylan 0.5 mg/mL, Mylan AB, Stockholm, Sweden). For eye-drops, 1 mg atropine sulfate (equivalent to 0.835 mg atropine) was administered in the conjunctival sac as a ophthalmic preparation (Isopto-atropin 1%, Alcon Nordic, Copenhagen, Denmark) in a total volume of 0.1 mL. Before atropine administration (time = 0), a pre-dose blood sample was drawn. Additional blood samples were drawn after atropine administration at minutes 5, 10, 15, 20, 40, 60, 120, 180, 240, 300, 360 and 420. For SPL constant rate infusion, 0.14 mg/h atropine sulfate (equivalent to 0.12 mg/h atropine) was administered.. A pre-dose blood sample was drawn. Additional blood samples were drawn after atropine administration at minutes 30, 60, 180, 240, 300, 420, 540, 660, 780 and 1440.

Blood-samples was drawn from the jugular vein contra-lateral to drug administration site and collected in heparinised tubes. Blood samples were centrifuged (1000 *g*, 4 °C) and the plasma was removed and frozen (− 70 °C) until analysis.

Part II: For the assessment of gastrointestinal effect of clinically relevant doses of topical treatment, 0.835 mg atropine was administered as 1 mg ophthalmic atropine sulfate (Isopto-atropin 1%, Alcon Nordic, Copenhagen, Denmark) in the conjunctival sac of one eye of each horse in a randomized blinded cross-over design. Two treatment protocols with active treatment plus control was evaluated with a 5 week wash-out period between study periods. The eye drops were administered in a total volume of 0.1 mL per administration. The following treatment protocols were used: A) a volume of 0.1 mL Isopto-atropine 1% every six hours for 2 days followed by once daily for 3 days alternated with administration of 0.1 mL 0.9% saline to mimic the treatment protocols B and C, B) a volume of 0.1 mL Isopto-atropine 1% every three hours for 2 days followed by twice daily for 3 days and C) for control, a total volume of 0.1 mL 0.9% saline was administered every third hour for 2 days followed by twice daily for 3 days.

### Analytical method for quantification of atropine plasma concentration

Quantitative analysis of atropine in plasma was carried out at the National Veterinary Institute (SVA) in Uppsala, Sweden. Internal standard (^2^H_5_-atropine (150 ng/mL, 50 μL)) was added to each plasma sample, calibrator or QC sample (100 μL). For protein precipitation, 100 μL of ice-cold acetonitrile were added and the samples were mixed (vortex) for 10 min and then centrifuged for 10 min at 11500 *g*. A part of the supernatant (50 μL) was diluted with water (100 μL) and 10 μL was injected into an ultra-high-performance liquid chromatography – tandem mass spectrometry (UHPLC-MS/MS) system. The chromatographic system consisted of an Acquity UPLC and the mass spectrometer was a Quattro Ultima Pt tandem quadrupole instrument with an electrospray ion source at positive potential (Waters Corporation, Milford, MA). The separation was carried out on an Acquity UPLC BEH C18 column (length 100 mm, I.D. 2.1 mm, particle size 1.7 μm) at 60 °C. The mobile phase was a mixture of (A) 10 mM ammonium formate in water and (B) 0.1% formic acid in acetonitrile and it was delivered as the following gradient: 20% B for 1.0 min, linear increase to 90% B during 1.0 min, 90% B for 1.0 min, linear decrease to 20% B during 0.1 min, 20% B for 1.9 min. The gradient time was 5.0 min in total and the flow-rate was 200 μL/min. The electrospray interface settings were: capillary voltage 1.5 kV and cone voltage 35 V. The desolvation temperature was set at 350 °C and the source block temperature was 150 °C. The desolvation gas flow was 650 L/h. The scan mode used was selected reaction monitoring (SRM) mode. The collision gas used was argon. The SRM transitions were m/z 290 → 124 for atropine (collision energy 28 eV) and m/z 295 → 124 for ^2^H_5_-atropine (collision energy 28 eV). The dwell time was 0.10 s. The reference substance for atropine and the internal standard ^2^H_5_-atropine were purchased from Toronto Research Chemicals (North York, ON; Canada). The chromatographic peak area ratio (analyte/internal standard) as a function of analyte concentration were used to calculate the calibration function using linear curve fit with a weighting factor of 1/x^2^. The calibration range was 0.05–500 ng/mL. The precision (relative standard deviation) was 1.3–14.9% and the accuracy was 95.9–104%.

### Pharmacokinetic model

Data analyses were carried out using Phoenix WinNonlin 8.0 (Pharsight Corporation St Louis, MO, USA). Data sets obtained from the three modalities of administration (IV, eye drops and infusion) were analyzed simultaneously using a Non-Linear Mixed Effect model (NLME). For infusion, plasma concentrations at the first sampling time (collected at about 0.5 h post-administration) was rather high and variable. According to the clinician having carried out the infusion, it is likely that an initial spurious atropine bolus dose occurred when activating the infusion pump. Thus, these first initial values were discarded from data analysis.

**Fig. 10:**
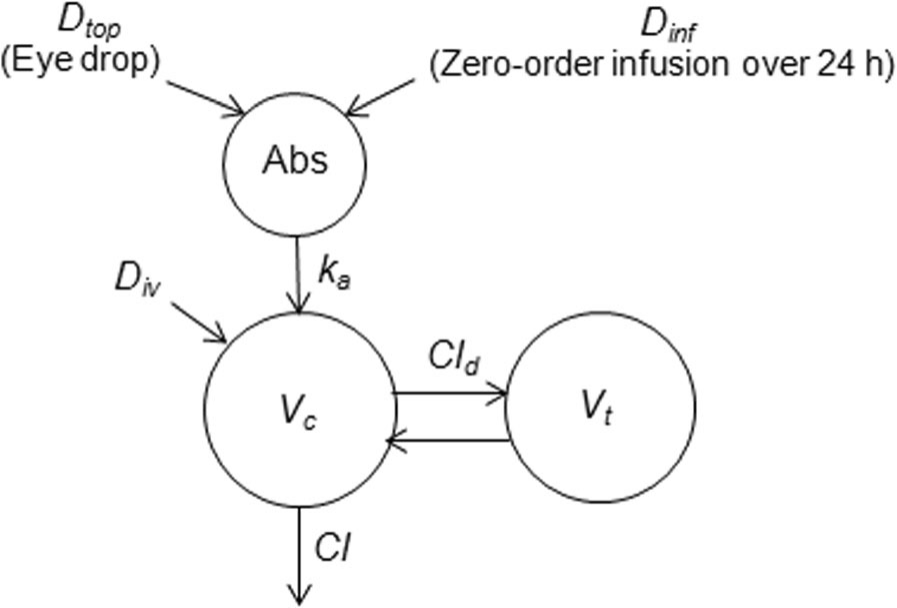
Schematic illustration of the two-compartment model used to characterize atropine concentration-time data. *D*_*iv*_, *D*_*top*_*, D*_*inf*_, *V*_*c*_, *V*_*t*_, *Cl, Cl*_*d*_ and *k*_*a*_*,*denote the dose administered intravenously (bolus), the dose administered topically in the eye either as drop (bolus) or an 24 h infusion (zero-order), the central and peripheral volume of distribution, clearance, inter-compartmental distribution clearance parameter and the absorption rate constant of atropine from the drug deposit in the eye to plasma, respectively

A two-compartment structural model (Fig. 10) was selected based on the Likelihood Ratio Test (LRT), the Akaike Information Criterion (AIC) and inspection of different diagnostic plots. Observations below LOQ were treated as censored, i.e. any positive value below 0.05 ng/mL was considered as plausible. Model was parametrized in terms of clearance and volume of distribution. Estimated parameters were the central (*V*_*c*_) and peripheral (*V*_*t*_) volumes of distribution, plasma clearance (*Cl*), inter-compartmental distribution clearance (*Cl*_*d*_), the absorption rate constant (*k*_*a*_) and bioavailability (F*drop* and F*infusion)* from the deposit to plasma after eye drops and infusion administration respectively. An ilogit transformation was used to estimate the bioavailability to ensure bioavailability estimates did not exceed 100%.

In a population model, the statistical model describing the inter-animal variability is included in the structural model. The inter-individual variation (IIV) for a given parameter was described using an exponential model of the form:

1 $$ {\theta}_{parameter\_i}={\theta}_{tv\_ parameter}\cdotp \mathit{\exp}\left({\eta}_i\right) $$

where *θ*_*parameter_i*_ is the value of theta for respective parameter in the i^th^ horse, *θ*_*tv*_*_*_*parameter*_ is the typical population value of the parameter (e.g. *V*_*c*_*, V*_*t*_*, Cl, Cl*_*d)*_ and *η*_*i*_ is the deviation from the corresponding theta population value associated to the *i*^*th*^ horse. The exponential model assumes a log-normal distribution of parameters, i.e. that the distribution of the etas (*η*_*i*_) is normal in the log-domain, with a mean of 0 and a variance ω^2^ where *η* ≈ *N*[0, *ω*^2^]. With the intention to report the IIV as coefficient of variation, eq. 2 was used for conversion of the variance terms (*ω*^2^) to a coefficient of variation (CV%).

2 $$ CV\left(\%\right)=100\times \sqrt{\mathit{\exp}\left({\omega}^2\right)-1} $$

Shrinkage of the random effects (ETA) toward the means was described as:

3 $$ shrinkage=1-\frac{\mathit{\operatorname{var}}\left({\eta}_r\right)}{\omega^2} $$

where *var(η*_*r*_*)* is the variance of the random effects. When shrinkage for eta was > 30%, it was considered that data were not able to estimate robustly this random component.

The residual model was an additive plus a multiplicative (proportional) model of the form:

4 $$ \mathrm{C}\left(\mathrm{t}\right)=f\left(\theta, Time\right)\times \left(1+{\varepsilon}_1\right)+{\varepsilon}_2 $$

with ε1, the multiplicative error term having a mean of 0 and a variance noted σ1

$$ \varepsilon 1\approx N\left(0,\sigma {1}^2\right) $$

ε2 the additive error term having a mean of 0 and a variance noted σ2

$$ \varepsilon 2\approx N\left(0,\sigma {2}^2\right) $$

The additive sigma was reported as its standard deviation noted with the same units as plasma concentration (μg/L) and the multiplicative sigma is reported as coefficient of variation.

As the same horses were enrolled in the three modalities of atropine administration, an interoccasion variability (IOV) measuring the intra-animal variability from treatment-to-treatment was incorporated in the model for plasma clearance. The IOV was of 10.32%. As this did not improve the overall fitting and as no individual prediction was in order, the IOV was not included in the final model.

Graphical inspection of Goodness-of-fit (GOF) plots to support the 2-comparmental structural model, the exponential model for the random component and the additive plus multiplicative model for the error submodel and the Akaike Information Criteria (AIC) were used to compare concurrent models.

Parameter estimation with the associated CV% as a measure of the precision of the estimation was based on minimizing an objective function value using maximum likelihood estimation. We used a Laplacian method that is appropriate when data below limit of quantification (BQL) are handled by the model. A bootstrap method was used to estimate precision parameters.

From the model parameters, different secondary parameters were estimated:

Bioavailability (from 0 to 1) was estimated from the corresponding ilogit typical values (eye drop and infusion) value as:

5 $$ F=\frac{EXP(ilogit)}{1+ EXP(ilogit)} $$

The terminal slope (*β*) of the atropine plasma concentration-time course was described as:

6 $$ \beta =0.5\bullet \left[\frac{Cl_d}{V_c}+\frac{Cl_d}{V_t}+\frac{Cl}{V_c}-{\left[{\left(\frac{Cl_d}{V_c}+\frac{Cl_d}{V_t}+\frac{Cl}{V_c}\right)}^2-4\frac{Cl_d}{V_t}\bullet \frac{Cl}{V_c}\right]}^{0.5}\right] $$

With *Cl*, *Cl*_*d*_, *V*_*c*_ and *V*_*t*_ as previously defined.

The terminal half-life (*t*_*1/2β*_) of the plasma atropine concentration-time course was described as:

7 $$ {t}_{1/2\beta }=\frac{\mathit{\ln}2}{\beta } $$

The apparent volume of distribution at steady stare (*V*_*ss*_) and the mean residence time (MRT) for atropine after an IV administration was computed as:

8 $$ {V}_{ss}={V}_c+{V}_t $$

9 $$ MRT=\frac{V_{ss}}{Cl} $$

Using the developed model, plasma concentration profiles corresponding to different atropine dosage regimen scenario were simulated (infusion at 2, 4, 8 μg per 24 h and drops (0.5 or 1 μg) at 1, 3 and 6 h intervals over 24 h. The model was solved using estimated typical value (PRED) i.e.by fixing ETA to 0 (no random component) for these simulations. In order to assess the consequence of the interanimal variability on the steady-state plasma concentration achieved by a 24 h infusion, a Monte Carlo Simulation was used to generate a population of 1000 horses using IPRED (ETA were as estimated) and the 90% prediction interval was computed, i.e. the range of plasma concentrations covering 90% of the horse population.

### Statistical analyses

Data from part II was subjected to statistical analyses. Statistical software was used to perform the data analyses (Minitab v19.2.0, 2019, State college, PA, U.S.A.). The Wilcoxon-signed-rank test was used to evaluate and compare heart rate, respiratory rate and fecal output between treatment groups and controls. The sign test was used to compare distributions of borborygmi-frequency between groups. *P*-values ≤0.05 were considered significant.

## Data Availability

The datasets generated during and/or analysed during the current study are available from the corresponding author on reasonable request.
